# A pipeline of programs for collecting and analyzing group II intron retroelement sequences from GenBank

**DOI:** 10.1186/1759-8753-4-28

**Published:** 2013-12-20

**Authors:** Michael Abebe, Manuel A Candales, Adrian Duong, Keyar S Hood, Tony Li, Ryan A E Neufeld, Abat Shakenov, Runda Sun, Li Wu, Ashley M Jarding, Cameron Semper, Steven Zimmerly

**Affiliations:** 1 Department of Biological Sciences, University of Calgary, Calgary, AB T2N 1 N4, Canada

**Keywords:** Bacteria, Genomes, Retroelement, Reverse transcriptase, Ribozyme

## Abstract

**Background:**

Accurate and complete identification of mobile elements is a challenging task in the current era of sequencing, given their large numbers and frequent truncations. Group II intron retroelements, which consist of a ribozyme and an intron-encoded protein (IEP), are usually identified in bacterial genomes through their IEP; however, the RNA component that defines the intron boundaries is often difficult to identify because of a lack of strong sequence conservation corresponding to the RNA structure. Compounding the problem of boundary definition is the fact that a majority of group II intron copies in bacteria are truncated.

**Results:**

Here we present a pipeline of 11 programs that collect and analyze group II intron sequences from GenBank. The pipeline begins with a BLAST search of GenBank using a set of representative group II IEPs as queries. Subsequent steps download the corresponding genomic sequences and flanks, filter out non-group II introns, assign introns to phylogenetic subclasses, filter out incomplete and/or non-functional introns, and assign IEP sequences and RNA boundaries to the full-length introns. In the final step, the redundancy in the data set is reduced by grouping introns into sets of ≥95% identity, with one example sequence chosen to be the representative.

**Conclusions:**

These programs should be useful for comprehensive identification of group II introns in sequence databases as data continue to rapidly accumulate.

## Background

**Figure 1:**
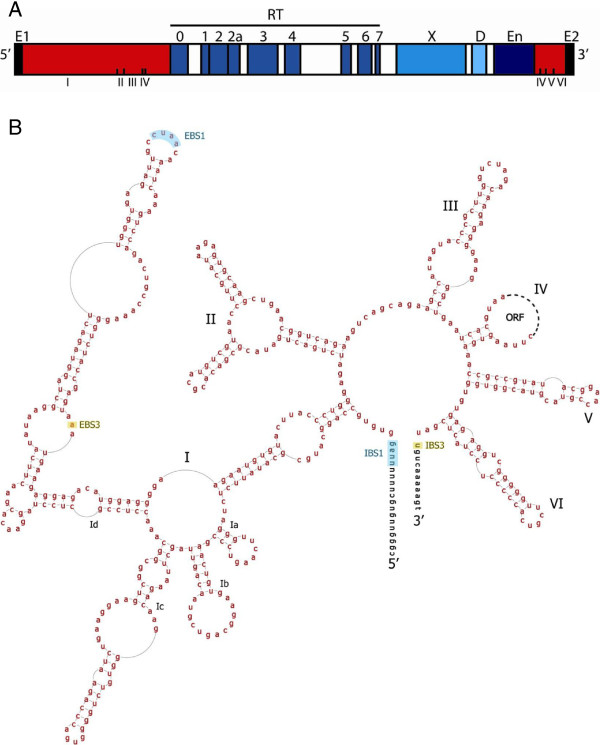
**Example group II intron structure. (A)** DNA structure of a group II intron. The intron RNA portion is denoted by red boxes, while conserved ORF domains are in blue. The IEP contains a RT (reverse transcriptase) domain, including conserved sub-domains (0, 1, 2, 2a, 3, 4, 5, 6, 7), an X domain, a D (DNA-binding) domain and an optional En (endonuclease) domain. Intron RNA domains are shown underneath in Roman numerals, and exon 1 and 2 sequences are in black. **(B)** An example group II intron RNA secondary structure (IIC). The intron sequence is depicted in red lettering, with exon sequences in blue and black. The ORF sequence is represented by the dotted loop in domain IV. IBS1/EBS1 and IBS3/EBS3 (blue and orange shading) represent base pairings between the intron and exons that help to define the intron boundaries during splicing. The sequence shown is for *B.h.*I1 of *Bacillus halodurans*.

Although not numerous, group II introns are found widely across the domains of life, being present in eubacteria, archaebacteria, and eukaryotic organelles [1-3]. The complete form of group II introns consists of two components: an RNA structure of ~500–800 nts and an intron-encoded protein (IEP) of ~400–700 amino acids. The RNA component is a ribozyme whose secondary structure is divided into six domains (DI–DVI), which fold into a tertiary structure capable of self-splicing (Figure 1) [4-6]. The IEP component is a multifunctional protein containing a reverse transcriptase (RT) domain with subdomains that are conserved across other RT families (subdomains 0, 1, 2, 2a, 3, 4, 5, 6, 7) (Figure 1) [7,8]. Downstream of the RT domain is domain X, which functions as the thumb domain of the RT, and is conserved in sequence among group II introns but not between group II introns and other types of RTs [9,10]. Immediately after domain X is a DNA binding domain (D), which is defined functionally but is not conserved in sequence [11]. Finally, many group II IEPs encode an endonuclease domain (En) at the C-terminus, which is required for retromobility of the introns that have it.

The retromobility mechanism of group II introns has been well-studied biochemically and genetically, and thorough descriptions can be found elsewhere [3,7,12,13]. Briefly, the ribonucleoprotein (RNP) particle formed during splicing recognizes a DNA target, and the intron RNA reverse splices into one strand of the DNA. The En domain cleaves the bottom strand, and the cleaved DNA is the primer for reverse transcription of the intron. Of relevance for this article, most group II introns have very high sequence selectivity for a long DNA target (~20–35 bp), which is known as a homing site. The homing site is sometimes in a conserved gene, in which case the intron boundaries can be defined or confirmed based on the interrupted ORF. However, many bacterial introns do not insert into conserved protein-coding sequences, making this criterion less useful in defining boundaries [14]. One sublineage, known as IIC or class C introns, inserts into the intergenic sequence after transcriptional terminator motifs with little sequence specificity [15].

Approximately 25% of eubacterial genomes and 10% of archaeal genomes contain at least one group II intron or a fragment of an intron [16,17]. Nearly all bacterial group II introns are of the complete retroelement form, containing both RNA and IEP components, although there are exceptions [17]. This is in contrast with group II introns in mitochondrial and chloroplast genomes, where there is an abundance of introns lacking IEPs in some organisms [18]. Because organellar introns are usually in housekeeping genes, the identification of organellar group II introns relies more heavily on the exon coding sequences.

Group II introns can be classified either phylogenetically according to their IEPs, or by features of their RNA secondary structures. Based on the IEPs, eight lineages of group II introns have been identified (ML, CL, A, B, C, D, E, F) [17,19]. By comparison, there are three classes of RNA structures (IIA, IIB, IIC) which are defined through their mechanisms of exon recognition [5,20,21]. Of the IEP classes, ML introns have a IIA RNA structure, CL introns have a IIB structure, and class C has a IIC structure. The remaining IEP classes are associated with variations of IIB secondary structures [17].

The IEPs of group II introns are easily identified through BLAST searches, because of their conservation. Incomplete IEPs are identified as truncations, in cases where RT subdomains or the X domain are missing or have premature stop codons. By contrast, the RNA component is not easily identified due to insufficient sequence conservation. For many introns, the RNA must be folded into a secondary structure in order to confidently predict the correct boundaries. Identifying the RNA boundaries is critical given that they specify the splice sites and define the protein translation product.

Across all group II introns, only domain V is highly conserved and reliably identified, while the remainder of the RNA has short and scattered segments of conservation [6]. For example, the 5′ and 3′ boundaries of group II introns have the short consensus sequences GUGYG and AY (Y = C,U), respectively, which are too short to unambiguously define boundaries. Taking into account the large number of truncated group II introns in genomes, it is often difficult to judge whether the sequence corresponds to a fragment of an intron, or whether the true boundary has been overlooked.

A useful fact in identifying RNA boundaries is that the ribozyme and IEP co-evolved (although there are exceptions) [22]. Thus, the IEP classes ML, CL, A, B, C, D, E, and F in effect define eight RNA secondary structure classes, which are fairly specific. For example, among class C (IIC) introns, the 5′ and 3′ boundary sequences have an expanded consensus sequence of 5′ GUNYGCCNRGCAUGG and CCUACYCGAU 3′ (R = A,G), which improves reliability in identifying the boundaries (Additional file 1: Figure S1). In many cases, using such class-specific consensus sequences, the boundaries can be confidently determined based on sequence alone, even if the entire secondary structure is not folded.

In the past we have compiled and catalogued group II introns in bacterial genomes in order to define structural types, distribution and spread, and to collect data for evolutionary analyses [16]. However, given the rapidly expanding databases of DNA sequence, a strategy of automation is required. Here, we present such a strategy with a set of eleven programs capable of collecting and analyzing group II intron sequences from GenBank.

## Implementation

**Figure 2:**
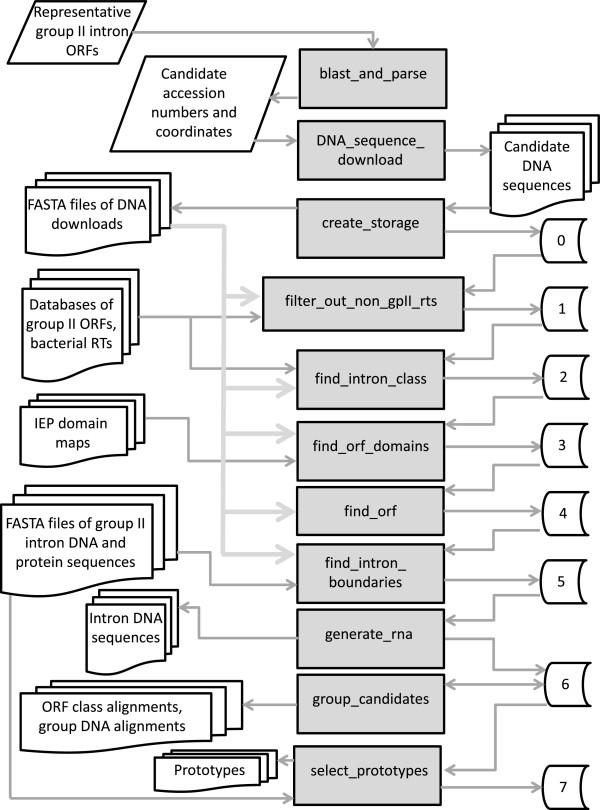
**Pipeline flowchart.** The pipeline proceeds through a series of steps in which data are collected and put into eight storage folders. Each storage folder feeds data into a subsequent program, which produces the next storage folder. The number of candidate introns decreases at each step, while more information accumulates for the smaller set of introns. To summarize the overall process briefly, a BLAST search identifies candidate IEPs in GenBank and DNA sequences are downloaded. RTs that are not IEPs are filtered out, and retained candidates are assigned to an intron class. ORF domains (0, 1, 2a, 2b, 3, 4, 5, 6, 7, X, En) are identified and ORF boundaries are annotated. The intron boundaries are then identified and an RNA structure is generated. Candidates with >95% similarity are grouped and a prototype from each group is identified.

The pipeline consists of eleven programs listed in Table 1 and diagrammed in Figure 2. The input to the program is a set of 22 group II intron ORFs that represent the known types of group II introns. In addition, there are five sets of data stored within the program that are used during the analyses: i) a set of 10 reference IEP sequences (one IEP per defined class/subclass) with domains defined for each (i.e., domains 0, 1, 2, 2a, 3, 4, 5, 6, 7, X, En); ii) a data set of 475 known group II introns with their defined DNA and IEP sequences; iii) a BLAST-searchable database of the 475 intron DNA sequences; iv) a BLAST-searchable database of the 475 IEP sequences; and v) a BLAST-searchable database of identified and categorized bacterial RTs (including group II introns, retrons, diversity-generating retroelements and other classified RT types) [23].

**Table 1:** Summary of programs

**Program**	**Steps**
blast_and_parse	• A tblastn search is done against NCBI’s ‘nr’ database with a set of representative group II intron ORF sequences as queries
	• A list of unique, non-overlapping candidate hits is assembled, along with accession number and coordinates
DNA_sequence_download	• The GenBank entry for each candidate DNA sequence is downloaded
	• Candidates are separated by taxonomic classification, with bacterial and archaeal candidates proceeding to the next step by default
create_storage	• Creates a FASTA file for each candidate’s DNA sequence
	• Creates storable files containing information about each candidate, to be used in later programs
filter_out_ non_gpII_rts	• A blastx search of candidate sequences is done against a local database of known, categorized bacterial RT sequences; candidate RTs whose closest relatives are not group II introns are separated out
find_intron_class	• A blastx search of candidate sequences is done against a local database of known and classified group II intron ORF sequences; based on the top matches, the ORF class is assigned, and the closest relative in the curated set is identified
find_orf_domains	• A blastx alignment is done between a candidate sequence and a representative IEP of the same class, whose IEP is mapped for the domains characteristic of group II introns
	• The domains present for each IEP are tabulated, and the candidate is categorized as having complete domains or missing domains; candidate sequences with complete IEP domains continue to be analyzed
find_orf	• A blastx alignment is done between each candidate sequence and its closest relative among curated group II introns
	• From the alignment, it is decided whether the candidate sequence contains frame shifts, premature stops or other problems within its IEP
	• If the ORF appears intact, then a predicted amino acid sequence is assigned
find_intron_boundaries	• Information on possible boundary positions is acquired using class-specific HMM profiles of boundary sequences
generate_rna_sequence	• Boundary sequence data are evaluated and the most probable intron boundaries are predicted, along with the complete sequence of the intron
	• Candidates with ambiguous boundaries are noted
group_candidates	• All ORF sequences assigned to a given class are aligned using ClustalW, and pair-wise distances are calculated using PROTDIST of the Phylip package
	• Sequences differing by less than 0.061 units are assigned to a group of 95% identity
	• For each group of 95% identity, the complete intron DNA sequence of each member is aligned using ClustalW
select_prototypes	• For each group of 95% identity, one candidate sequence is selected as the prototype, or representative of the group

To briefly summarize the process, the 22 representative IEPs are used as queries to search GenBank for candidate IEPs. Detected DNA sequences are downloaded along with 3 kb of flanks upstream and downstream of the IEP. The putative IEPs are screened to retain only bacterial and archaeal sequences, and to exclude RTs that are not group II introns. Each candidate intron is tentatively assigned to a class (ML, CL, A, B, C, D, E, F, unclassified, undefined). The ORF domains present in each IEP are determined and IEPs that are judged to be intact are assigned an amino acid sequence. Intron RNA boundaries are predicted based on class-specific sequence profiles for 5′ and 3′ boundaries. To eliminate redundancy in the final set of introns, introns are grouped into sets of ~95% identity and one intron in each set is selected as the representative, or prototype.

The output of the programs is in two folders, one containing data for the complete set of full-length and presumably functional introns, and the second containing data for a reduced set of non-redundant introns (<95% identity); however, if an intron in a 95% group is from a different species, it is included as a unique intron despite the sequence redundancy. In addition to this final output, data can be accessed at each step of the pipeline, and it is possible to write the accumulated data to more reader-friendly files using flags.

The programs run on a 64-bit LINUX system with internet access. Programs that must be installed on the computer include the following: HMMER2 (2.3.2, available from http://hmmer.janelia.org/software/archive; the path to the HMMER2 programs should be an environment variable $HMMER2), HMMER3 (3.0, available from http://hmmer.janelia.org/software; the path to the HMMER3 programs should be an environment variable $HMMER3); the BLAST + suite (2.2.26) from NCBI (ftp://ftp.ncbi.nlm.nih.gov/blast/executables/blast+/2.2.26/); CLUSTALW (2.1 /ftp://ftp.ebi.ac.uk/pub/software/clustalw2/2.1/); and PHYLIP (3.69/ http://evolution.genetics.washington.edu/phylip/getme.html). Other modules such as BioPerl utilities are embedded within the executable files.

The program runs by command lines. Typical commands are specified in Additional file 2: Table S1, along with optional settings. Executable program files are available as supplemental files or at our web site (http://webapps2.ucalgary.ca/~groupii/index.html) where updates will become available.

## Results and discussion

### Detailed description of programs

#### *blast_and_parse*

The initial input is a collection of 22 ORFs representing known classes of group II introns (ML, CL, A, B, C, D, E, F, and unclassified introns). For each ORF, the program connects to NCBI and searches GenBank for sequence matches using TBLASTN (protein query vs. translated DNA database), and using the non-redundant database with an E value cut-off of 1x10^-20^. The use of TBLASTN is important because it detects unannotated IEPs and the output format gives genomic coordinates of the ORF matches. If desired, the user can choose to use another set of ORFs as queries, or search another GenBank database (e.g., whole genome shotgun database), or specify a different E value cut-off (Additional file 2: Table S1). The result of the searches is a set of 22 files in standard BLAST output format, one for each TBLASTN search. The 22 text files are parsed for the genomic boundaries of each HSP (high-scoring pair), and the results are combined into one collection of accession numbers and coordinates, with duplicates eliminated during the pooling step. The output files and folders produced by all programs are listed in Additional file 2: Table S2.

#### *DNA_sequence_download*

For each accession number and coordinates, the DNA sequence is downloaded from GenBank in standard GenBank format along with 3 kb of flanking sequence both upstream and downstream of the HSP match (~8–10 kb total). The DNA sequence is downloaded in the orientation that encodes the IEP on the forward strand. Taxonomic information is collected from the GenBank entry, which allows the files to be divided into five sets: “bacteria & archaebacteria”, “eukaryotes”, “mitochondria”, “chloroplasts”, and “other.” For each of the five sets, the texts of all of the GenBank entries are concatenated and saved as a single text file. The “bacteria_and_archaea” set is used by default in subsequent programs, but it is possible to input other folders or to introduce other folders as input of data in subsequent programs.

#### *create_storage*

A folder is created, “storage_new”, which over the course of the pipeline will contain a series of accumulated information. At this point, the folder “0” is created, but in subsequent programs storage folders 1–7 are generated, each containing progressively more data as further analyses are done (Figure 2). Folder 0 contains the following information: Accession numbers, coordinates of the HSPs, length of the HSPs, the strand encoding the IEP, conversion data to allow correspondence with the original GenBank numbering, and the full GenBank entry. Information in the storable files is formatted for subsequent use in the programs, but if desired, the data can be obtained in an organization that is more reader-friendly using optional flags (Additional file 2: Table S1).

#### *filter_out_non_gpII_rts*

A significant challenge in assembling a comprehensive set of group II introns is that there are RTs in bacteria that give BLAST matches to IEPs but are not group II introns. One way to eliminate them is to use a more stringent E value cut-off in the initial BLAST search; however, the stringency also eliminates some true group II introns that are not closely related to those already identified. Complicating this scenario is the fact that some RT types are very closely related to group II introns and presumably derived from them, yet do not have associated RNA structures (e.g., CRISPR-associated RTs) [23]. Therefore, it is challenging to comprehensively collect group II intron RTs while excluding non-group II intron RTs.

Our strategy to deal with this issue is to do multiple BLAST searches with relatively low stringency, using as queries a variety of intron subtypes, and then combining the results and subtracting out the non-group II introns. We use an E value cut-off of 1x10^-20^ in the BLAST search; relaxing the stringency mainly increases the number of short fragments of group II introns. Previously, we compiled a list of RT types in bacteria, including group II introns, retrons, diversity-generating retroelements and a variety of additional uncharacterized RTs [23]. Using this list, a BLAST-searchable database of their sequences was produced. To identify the non-group II introns in the set of downloaded candidate intron sequences, each candidate IEP sequence is searched against the local database of RT types. Candidates are considered probable group II introns if the top three matches are also group II introns; they are considered possible group II introns if one or two of the top three matches are group II introns; and they are considered not to be group II introns if the three top matches are not group II introns. The stringency of this test can be adjusted using flags (Additional file 2: Table S1). By default, only probable group II introns continue to the next stage of analysis.

#### *find_intron_class*

The phylogenetic class for each candidate IEP is next assigned, using a BLASTX search (translated nucleotide query vs. protein database) in which a local database of 475 group II intron IEPs is searched using as a query each candidate DNA sequence. If the three best matches are of the same class, the candidate is taken to belong to that class as well. Classes assigned are ML, CL, A, B, C, D, E, F, undefined, or unclassified. “Undefined” denotes that the top three hits are not of the same class, whereas “unclassified” means that the top three hits are to introns designated “unclassified”). The BLAST results are also used to identify the closest intron relative of each candidate among the 475 curated group II introns, which will be used later in the pipeline.

#### *find_orf_domains*

To determine which ORF domains are present in each candidate sequence, a set of nine reference IEPs is used, with one IEP per class (ML, CL1, CL2, A, B, C, D, E, F). For each reference IEP, one conserved amino acid in each domain (0, 1, 2, 3, 4, 5, 6, 7, X, En) is chosen as a proxy for the presence of that domain. A BLASTP alignment is made between each candidate sequence and the reference intron for its class. If a given amino acid proxy is present in the pair-wise alignment, then the IEP is judged to contain that domain (the specific amino acid does not have to be identical in the two sequences, but must be present in the pairwise alignment). This step determines whether the candidate contains all expected IEP domains, or is truncated and missing expected domains. Candidate sequences are sorted into folders according to the ORF characteristics. The IEP is assigned as “normal” if all domains are present once in the sequence, “missing domains” if one or more expected domains are absent, “extra domains” if one or more domains are present more than once (e.g., in a twintron), and “missing and extra domains” if one or more domains are missing and one or more domains are present more than once (i.e., a complex intron arrangement). By default, only complete IEPs continue to the next step in the pipeline.

#### *find_orf*

The presence of all domains does not necessarily mean that the IEP is intact, because there may be frame shifts, premature stops, or other problems within the ORF. While predicting IEP function is inherently speculative, we use the criterion of a single, uninterrupted alignment between a candidate IEP and its closest, curated IEP relative. To identify such proteins, a pairwise BLASTX alignment is done between each candidate DNA sequence and its closest IEP relative. A putatively intact ORF is indicated by the absence of stop codons, by the absence of insertions (gaps), and also by the alignment consisting of a single HSP (i.e., frame shifts or large indels would cause the alignment to be in more than one HSP). If no problems are identified, then the amino acid sequence of the IEP is assigned. Because many GenBank sequence files are not annotated, and also because N-termini are often misannotated in GenBank, the IEP sequences are assigned based on the amino acid sequences of the closest relative in the set of 475 curated introns. The start codon is chosen according to the start codon of the closest annotated group II intron sequence and the presence of an upstream Shine-Dalgarno sequence.

#### *find_intron_boundaries*

As described above, group II intron boundaries are not identified reliably on the basis of a generalized consensus sequence; however, class-specific sequence patterns give greater accuracy. Taking this into account, hidden Markov model (HMM) profiles have been made for the boundary sequences of each class, and are used to search for potential 5′ and 3′ termini. Simple consensus sequences of the boundary sequences for the classes are shown in Additional file 1: Figure S1 in order to illustrate the class-specific differences in sequence patterns. Each candidate DNA sequence is searched using the HMM profile of its own class; or if a sequence is “unclassified” or “undefined” it is searched with the profiles of all classes. Both HMMER2 and HMMER3 search programs [24] are used because it was found, for unknown reasons, that each program works better for certain intron sequences; hence, both programs are run, and the best score is used. For each candidate sequence, the DNA matches to the HMM profile are ranked, and the candidate sequence is placed into one of four subfolders to denote that it has matches for “both 5′ and 3′ boundaries”, “only 3′ boundary”, “only 5′ boundary” or “no boundaries.” Each subfolder is further subdivided according to probability scores of the HMM matches (high, medium, low) (Additional file 2: Table S2).

#### *generate_rna_sequences*

The profile matches for each intron candidate are evaluated in order to judge the most probable intron boundaries out of the compiled possibilities. A full-length, intact intron is indicated when there is only one plausible 5′ and one 3′ boundary, and they are in the correct order and separated by a reasonable distance. If these conditions are met, then the full intron sequence is generated based on those boundaries. Candidate introns are sorted into folders of “have boundaries” and “ambiguous intron boundaries”. By default, only introns with “have boundaries” continue to the following programs.

#### *group_candidates*

Because group II introns are mobile DNAs, there are sometimes many identical or nearly identical intron sequences in a genome or in different GenBank entries. In order to eliminate the redundancy, introns are grouped into sets of introns of >95% identity. To do this, each phylogenetic class of IEPs is analyzed separately. The ORF sequences are aligned across the region corresponding to domains 0 to X, using ClustalW [25]. The alignment is analyzed using PROTDIST of the Phylip package to generate pairwise distances and a crude tree [26]. Candidate sequences that are less than 0.061 units apart (empirically determined to correspond to ~95% identity) are assigned to the same group of 95% identity. The phylogenetic analysis based on the automatic alignment is obviously crude, but sequences of >95% identity should be correctly identified.

#### *select_prototypes*

Finally, one intron in each group of 95% identity is chosen to be the representative, or prototype, unless there are multiple species within the group, in which case each species is assigned a prototype as well. The final output goes to a series of folders and files listed in Additional file 2: Table S2. Using the “write” flag gives the same information but in a somewhat more user-friendly organization.

### Efficacy, completeness and accuracy

The described programs successfully collect and download sequence information from GenBank, sort them into classes, identify the ORF and ribozyme components and boundaries, and create a non-redundant list with <95% identities. At each step of the pipeline, an aspect of the introns is examined and introns appearing not to be full-length and/or functional are set aside, with only intact sequences going forward. This produces a steadily smaller set of introns, with more information accumulated at each step for the introns that remain. As of July 2013, 3,191 non-redundant HSPs were identified initially as candidate group II introns in the “blast_and_parse” step, while at the end of the pipeline 572 of these were identified as prototypes. A detailed account of the segregation of the 3,191 sequence files into different categories over the course of the programs is listed in Additional file 2: Table S2. At each step, the sequences that are set aside can be examined or analyzed if desired, to find missed introns for example, or to collect intron sequences having certain characteristics.

To measure the accuracy and completeness of the pipeline, a set of 513 introns present in the initial set of 3,191 downloaded sequences was followed through the process. At the end of the pipeline, 451 of 513 (88%) were included in the set of prototypes, which by default includes only “high probability” introns. If introns in the categories of medium and low probability are included as well, then 479 of 513 (93%) were identified. If one considers only the single step that identifies boundaries for introns using the HMM profiles (“find_intron_boundaries”), and take into account the best predictions regardless of the probability estimates (high, medium, low), then 477 out of 492 (97%) introns analyzed by the program have the correct boundaries.

A substantial portion of the missed introns (21 of 513 sequences, 4%) corresponds to the set of twintrons (introns nested within other introns) and other candidate DNA sequences containing more than one intron copy. These sequences were excluded at the “find_orf” stage of the pipeline. Their removal serves to prevent errors in subsequent steps due to multiple introns being present in a sequence being analyzed; however, it has the consequence of excluding sequences with more than one intron in the 8–10 kb of downloaded sequence. These introns, however, can be recovered from the “multiple ORF locations” folder generated by the “find_orf” program, and manually examined to determine their exact organizations.

A major reason for the remaining missed introns is the specificity of some of the HMM profiles. Sequences considered “unclassified” were screened with the profiles of other classes, which not surprisingly reduced specificity and success. In addition, boundary profiles for Classes E and F were constructed from a relatively small number of introns and the prediction success was notably lower than for profiles of other classes. Overall, the boundaries for Class E, F and unclassified introns were correctly predicted with “high probability” for only 36 of 67 introns (54%) by the program “find_intron_boundaries.” However, including the single, best “low” or “medium” probability prediction resulted in 58 of 67 introns (87%) with correct boundaries.

In the future, the HMM profiles can be improved substantially by increasing the number of E and F introns in the alignments, and by identifying additional classes from the “unclassified” introns, when enough examples are available to define a group and corresponding sequence pattern. An alternative strategy for identifying introns not belonging to established classes is to manually examine the sets of failed sequences after the “find_intron_boundaries” program. These sequences have intact IEP sequences but lack predicted 5′ and/or 3′ boundaries. Several of them appear to be bona fide introns that belong to new classes (unpublished data), which will be reported elsewhere.

Another limitation of the boundary prediction algorithm is illustrated by two sets of introns that have insertions or extensions at either the 5′ or 3′ termini. An unusual subset of CL1 (IIB1) introns has a 5′ insertion near the start of the intron, which can be hundreds of nucleotides long [27]. A second set of introns belonging to Class B has a 3′ extension located after domain VI, with splicing occurring ~50–70 nucleotides downstream of the end of domain VI [28]. For both of these intron types, the programs failed to locate the correct termini and instead identified suboptimal 5′ or 3′ boundaries at the location typical for other introns. For specialized intron variants such as these, it may be possible to computationally predict the unusual boundaries, but the variants have to be defined first, and then the pattern can be searched for.

Finally, it should be noted that introns without IEPs will be missed by this algorithm, as well as introns that encode an IEP not belonging to the RT family, such as the LAGLIDADG-encoding intron in *Thiomargarita namibiensis*. However, these introns do not appear to be common in bacteria [29].

## Conclusions

This suite of programs allows for comprehensive, automated detection of group II introns from GenBank, and provides an alternative to manual curation of group II introns amidst the rapidly expanding sequence databases. While not without limitations, the programs give effective tools for handling group II intron sequences and determining the scope and diversity of group II intron sequences present in bacterial genomes. Future updates to the programs, as well as larger libraries of curated group II introns, will improve the performance of the pipeline over time.

## Availability and requirements

• Project Name: Group II intron identification pipeline

• Project Home Page: http://webapps2.ucalgary.ca/~groupii/index.html

• Operating System: Linux 64-bit

• Other requirements: HMMER2 2.3.2, HMMER3 3.0, BLAST + Suite 2.2.26, ClustalW 2.1, PHYLIP 3.69

• License: None

• Restrictions of use by non-academics: None

## Abbreviations

D: DNA binding domain; En: Endonuclease domain; HMM: Hidden Markov model; IEP: Intron-encoded protein; RNP: Ribonucleoprotein; RT: Reverse transcriptase.

## Competing interests

The authors declare that they have no competing interests.

## Authors’ contributions

Programs were written by MA, MAC, AD, KSH, TL, RAEN, AS, and RS, and were devised by these authors and SZ. Output generated by the programs were evaluated by LW and AMJ. The manuscript was written by SZ and CJS with input from all authors. All authors read and approved the final manuscript.

## Supplementary Material

Additional file 1: Figure S1 Class-specific consensus sequences for 5′ and 3′ intron boundaries. Click here for file

Additional file 2: Table S1 Input commands and options for each program. **Table S2.** Output of each program. Click here for file
