# Supplementary material for: A pipeline of programs for collecting and analyzing group II intron retroelement sequences from GenBank
Source: Mob DNA. 2013 Dec 20;4:28. doi: 10.1186/1759-8753-4-28 (PMC4028801; doi:10.1186/1759-8753-4-28)
Supplement: Additional file 1: Figure S1 — Class-specific consensus sequences for 5′ and 3′ intron boundaries. [file 1759-8753-4-28-S1.doc]

Class |--5’ boundary sequence and start of domain 1--------//-|------------domain 5--------------|-|-------------------------------domain 6 and 3’ boundary sequence--|

A GUGyGCCynG--[16-38nt]--rUnUGnCUGGAAU-----------------//-GAGCCGnAUGCGCU-GAAA-GGUGCACGUnCGGUUC-GGGGAGGAGArGC---------------------[9-24nt]---------GCyUCUU-A-CUC-yAC

B GUGCGAnACGUU--[36-119nt]--AACUrnynnnyyGA-------------//-ACGCCGUrUGnGGn-GAAA-nUCnCAyGyACGGUGU-GrAGyGGGGGAAAA-------------------[15-72nt]----AAAnnyUUACCU-A-UCryUAU

C GUnyGCCnrGCAUGG--[2-83nt]--CUnAnnnGG-----------------//-CCGCCGUrUrCG---GAnC---CGyAyGynnGGUGG-UGUGrGrGG------------------------[15-42nt]-------------CCU-A-CyC-GAU

D GUGUGCCnyR--[2-51nt]--GCUnyrnnrrAGAUG----------------//-GAGCCGnrUGAnnn-GAGA-GnnUCACGyACGGUUC-UGnGAGrGnCynnnG-------------GUGA------------ArUUCCyyyGGyCU-A-CUCr-nC

E GUGCGCCrrGnnArrnrnryrnn--[0-47nt]--rnnyArnnGGUGnAA---//-GAGCCGnrUGCGG--nAAnn-CyGCAnGynCGGnUC-UGUGnGGGGGn-----------------------[3-96nt]----------rnnyCU-A-CCnCGAy

F GUryGyCnnG--[3-51nt]--nynnny--[0-6nt]--nnnnyyAGyrGG--//-rAGCCGnrUGCGG--GAAAA-CCGCAyGynCGGUUy-GAyGAGGGrGrrnn-------------------[10-102nt]---------yyyyCn-A-CyCU-AC

CL1 GUGCGACnnG--[0-8nt]--AAGyyGynynnn--[0-36]--nnryUGyy--//-GAGCCGUAUGnnGn-GAAA-nUynCAyGynCGGUUC-UUAGGGGrGnrnnnnnyr----------GyAA--------------yrnnnnnynCyU-A-CCCG-AC

CL2 GUGCGAUUCGUU--[52-158nt]--AACUnnnnnnyynUGnnGGU-------//-GAGCnGUrUGAnGn-GAAA-nUyUCAyGynCnGUUy-UGAAGnnGAGynrrrrrG--[0-7]---GyGA----[0-7]-----yyyyyyynryyn-A-nyyyAAy

ML GUGCGCCCnUAA-----------------------------------------//-GAGCCGnAUrCnyy-GArA-GnnGyAyGUnCGGUUC-GGnGGGGRGnnnnnrr---[1-27nt]-GNAA---[8-28nt]------------CyU-A-yCCy-AC

Additional file 1: Figure S1. Class-specific consensus sequences for 5’ and 3’ intron boundaries. Specific bases represent >50% identity for the position, while “r” and “y” represent >75% purines and pyrimidines, respectively; “n” is any nucleotide. The double slash “//” denotes the sequence discontinuity between the 5’ and 3’ sequences of the intron. Numbers in brackets are the number of nucleotides present in individual intron sequences but not shown in the consensus sequence. Consensus sequences were made from the following number of individual sequences: 7 (A), 50 (B), 168 (C), 50 (D), 43 (E), 31 (F), 85 (CL1), 52 (CL2), and 46 (ML). The CL1 introns with large insertions near their 5’ ends were excluded from the consensus, as were the Class B introns with 3’ extensions (domain 7) (see main text).
