# Supplementary material for: A pipeline of programs for collecting and analyzing group II intron retroelement sequences from GenBank
Source: Mob DNA. 2013 Dec 20;4:28. doi: 10.1186/1759-8753-4-28 (PMC4028801; doi:10.1186/1759-8753-4-28)
Supplement: Additional file 2: Table S1 — Input commands and options for each program. Table S2. Output of each program. [file 1759-8753-4-28-S2.docx]

**Additional file 2: Table S1. Input commands and options for each program**

| **blast_and_parse** | | |
| --- | --- | --- |
| Standard command | ./blast_and_parse -b ../system_new/blast_alignments -u ../system_new/unique_candidate_hits.txt | |
| Usage | ./blast_and_parse -b[lastout] DIR1 -u[niqueout] FILE1 [options] | |
| Mandatory settings | -b[lastout] DIR1 | Creates the output directory DIR1 which contains the results of the blast search |
|  | -u[niqueout] FILE1 | Creates the output file FILE1 which lists the unique candidate hits to download |
| Optional settings | -i[nputIEPs] FILE2 | Uses the file FILE2 as query protein sequences in the tblastn search |
|  | -d[atabase\|b] STRING | Sets the GenBank nucleotide database for the tblastn search to be STRING, default is 'nr' database, other available databases are specified at the GenBank web site |
|  | -e[value] NUM | Sets the tblastn Expect threshold to be NUM, default is '1e-20' |
|  | -f[lanklen] INT | Sets the flank length to be INT, greater than 0, default is '3000' bps; if two HSPs lie within the flank length, then only one DNA sequence will be downloaded for them both, containing both HSPs |
| Example of optional usage | ./blast_and_parse -b ../system_new/blast_alignments -u ../system_new/unique_candidate_hits.txt -i ../start_off_files/Representative_Gp_II_Intron_ORFs.txt -d wgs -e 1e-10 -f 5000  Result: searches the whole genome shotgun (wgs) database of GenBank with protein queries from the file ../start_off_files/Representative_Gp_II_Intron_ORFs.txt, using an e value of 1e-10, with a flank length setting of 5000 bp upstream and downstream | |
| **DNA_sequence_download** | | |
| Standard command | ./DNA_sequence_download -u ../system_new/unique_candidate_hits.txt -d ../system_new/sequences.txt -fi ../system_new/candidate_Genbank_files | |
| Usage | ./DNA_sequence_download -u[niquein] FILE1 -d[ownload] FILE2 -fi[lter] DIR1 [options] | |
| Mandatory settings | -u[niquein] FILE1 | A path to a file FILE1 that contains download request information |
|  | -d[ownload] FILE2 | Creates the output file FILE2 that contains the downloaded GenBank entries concatenated together into one file |
|  | -fi[lter] DIR1 | Creates the output directory DIR1 that contains GenBank entries separated into taxonomic groupings |

| Optional settings | -fa[iled] FILE3 | Creates the output file FILE3 that contains the GenBank entries whose taxonomic grouping could not be properly parsed |
| --- | --- | --- |
|  | -fl[anklen] INT | Sets the downloaded flank length to be INT, greater than 0, default is '3000' bps (i.e., the DNA sequence of the IEP is downloaded along with 3000 bp upstream and 3000 bp downstream) |
| Example of optional usage | ./DNA_sequence_download -u ../system_new/unique_candidate_hits.txt -d ../system_new/sequences.txt -fi ../system_new/candidate_Genbank_files -fa ../system_new/failed_to_parse.txt -fl 5000  Result: Downloads GenBank entry with 5000 bp flanks upstream and downstream, writing the entries with ambiguous taxonomy to the file ../system_new/failed_to_parse.txt | |
| **create_storage** | | |
| Standard command | ./create_storage -g ../system_new/candidate_Genbank_files/bacterial_and_archaea.txt -s ../storage_new/0 -f ../system_new/FASTA | |
| Usage | ./create_storage -g[enbank] FILE1 -s[torable] DIR1 -f[asta] DIR3 | |
| Mandatory settings | -g[enbank] FILE1 | A path to a file FILE1 that contains downloaded GenBank entries |
|  | -s[torable] DIR1 | Creates the output directory DIR1 that stores files for each candidate sequence |
|  | -f[asta]\|F[ASTA] DIR2 | Creates the output directory DIR2 that contains FASTA formatted nucleotide sequences for each candidate |
| **filter_out_non_gpII_rts** | | |
| Standard command | ./filter_out_non_gpII_rts -si ../storage_new/0 -so ../storage_new/1 -f ../system_new/FASTA/ | |
| Usage | ./filter_out_non_gpII_rts -si\|storablein DIR1 -so\|storableout DIR2 -f[asta]\|F[ASTA] DIR3 [options] | |
| Mandatory settings | -si\|storablein DIR1 | A path to a directory DIR1 that stores the blast-searchable database used to filter out the non-group II intron RTs |
|  | -so\|storableout DIR2 | Creates the output directory DIR2 that stores RTs sorted into categories of “Probably a Group II Intron” and “Probably Not a Group II Intron” |
|  | -f[asta]\|F[ASTA] DIR3 | A path to a directory DIR3 that contains FASTA formatted nucleotide sequences for each candidate |

| Optional settings | -w[ritetofile] DIR4 | Creates the directory DIR4 that contains human readable output of sorted candidate RTs |
| --- | --- | --- |
|  | -c[utoff] INT | The number of best hits required for a candidate to be classified as 'Probably a Group II Intron'; default is three (i.e. the three top hits are group II intron IEPs) |
| Example of optional usage | ./filter_out_non_gpII_rts -si ../storage_new/0 -so ../storage_new/1 -f ../system_new/FASTA/ -w ../readable/filter_rts -c 5  Result: Filters out non-group II intron candidates, with human readable output printed to ../readable/filter_rts. To be considered “Probably a Group II Intron” the top five hits must be known group II introns. | |
| **find_intron_class** | | |
| Standard command | ./find_intron_class -si ../storage_new/1/Probably\ Group\ II\ Intron/ -so ../storage_new/2/ -f ../system_new/FASTA/ | |
| Usage | ./find_intron_classes -si\|storablein DIR1 -so\|storableout DIR2 -f[asta]\|F[ASTA] DIR3 | |
| Mandatory settings | -si\|storablein DIR1 | A path to a directory DIR1 that stores the blast-searchable reference IEP files used to assign ORF classes |
|  | -so\|storableout DIR2 | Creates the output directory DIR2 that stores candidate files in subdirectories sorted by assigned ORF classes |
|  | -f[asta]\|F[ASTA] DIR3 | A path to a directory DIR3 that contains FASTA formatted nucleotide sequence for each candidate |
| Optional settings | -w[ritetofile] DIR4 | Creates the directory DIR4 that contains human readable output of candidate sequences sorted by ORF class |
|  | -c[utoff] INT | The number of best hits required for a candidate to be in a class; the default is 3 (i.e., the three top hits must belong to that class) |
| Example of optional usage | ./find_intron_class -si ../storage_new/1/Probably\ Group\ II\ Intron/ -so ../storage_new/2/ -f ../system_new/FASTA/ -w ../readable/Classes -c 5  Result: Assigns candidate sequences into classes, with human readable output printed to ../readable/Classes. Sequences must match the same class within the top 5 hits to be assigned to that class. | |
| **find_orf_domains** | | |
| Standard command | ./find_orf_domains -si ../storage_new/2/all/ -so ../storage_new/3 -f ../system_new/FASTA/ | |
| Usage | ./find_orf_domains -si\|storablein DIR1 -so\|storableout DIR2 -f[asta]\|F[ASTA] DIR3 | |
| Mandatory settings | -si\|storablein DIR1 | A path to a directory DIR1 that stores files that define IEP domains for each class |
|  | -so\|storableout DIR2 | Creates the output directory DIR2 that stores files sorted into subdirectories based on completeness of IEP domains |
|  | -f[asta]\|F[ASTA] DIR3 | A path to a directory DIR3 that contains FASTA formatted nucleotide sequences for each candidate |
| Optional settings | -w[ritetofile] DIR4 | Creates the directory DIR4 that contains human readable output of assigned IEP domains for each candidate |
| Example of optional usage | ./find_orf_domains -si ../storage_new/2/all/ -so ../storage_new/3 -f ../system_new/FASTA/ -w ../readable/Domains -p ../IEP_Domain_Maps  Result: Assigns IEP domains for each candidate, and writes the results to ../readable/Domains. | |
| **find_orf** | | |
| Standard command | ./find_orf -si ../storage_new/3/Normal\ ORF\ Domains/ -so ../storage_new/4 -f ../system_new/FASTA | |
| Usage | ./find_orf -si\|storablein DIR1 -so\|storableout DIR2 -f[asta]\|F[ASTA] DIR3 | |
| Mandatory settings | -si\|storablein DIR1 | A path to a directory DIR1 that stores files that are used as the input sequences for assigning ORF functionality |
|  | -so\|storableout DIR2 | Creates the output directory DIR2 that stores files sorted into subdirectories from assigning ORF functionality |
|  | -f[asta]\|F[ASTA] DIR3 | A path to a directory DIR3 that contains FASTA formatted nucleotide sequences for each candidate |
| Optional settings | -w[ritetofile] DIR4 | Creates the directory DIR4 that contains human readable output from assigning candidate sequences ORF functionality |
| Example of optional usage | ./find_orf -si ../storage_new/3/Normal\ ORF\ Domains/ -so ../storage_new/4 -w ../readable/ORF  Result: Assigns candidate sequences ORF functionality, and human readable output is printed to ../readable/ORF. | |
| **find_intron_boundaries** | | |
| Standard command | ./find_intron_boundaries -si ../storage_new/4/Apparent\ Functional\ ORF -so ../storage_new/5 -f ../system_new/FASTA/ | |
| Usage | ./find_intron_boundaries DIR1 -so\|storableout DIR2 -f[asta]\|F[ASTA] DIR3 | |
| Mandatory settings | -si\|storablein DIR1 | A path to a directory DIR1 that stores files that are used as the input sequences for assigning intron boundaries |
|  | -so\|storableout DIR2 | Creates the output directory DIR2 that stores files sorted into subdirectories from assigning intron boundaries |
|  | -f[asta]\|F[ASTA] DIR3 | A path to a directory DIR3 that contains FASTA formatted nucleotide sequences for each candidate |
| Optional settings | -w[ritetofile] DIR4 | Creates the directory DIR4 that contains human readable output from assigning candidate sequences intron boundaries |
| Example of optional usage | ./find_intron_boundaries -si ../storage_new/4/Apparent\ Functional\ ORF -so ../storage_new/5 -f ../system_new/FASTA/ -w ../readable/Boundaries  Result: Acquires information about possible boundaries of candidate sequences, and prints the data to ../readable/Boundaries. | |
| **generate_rna_sequences** | | |
| Standard command | ./generate_rna_sequences -si ../storage_new/5/Both\ 5\'\ and\ 3\'\ Boundaries/all/ -so ../storage_new/6 -o ../system_new/Class_RNA_and_DNA_Sequences | |
| Usage | ./generate_rna_sequences -si\|storablein DIR1 -so\|storableout DIR2 -o[d\|utdir] DIR3 | |
| Mandatory settings | -si\|storablein DIR1 | A path to a directory DIR1 that stores files that are used as the input sequences for assigning the intron RNA sequence |
|  | -so\|storableout DIR2 | Creates the output directory DIR2 that stores files sorted into subdirectories from assigning the intron RNA sequence |
|  | -o[d\|utdir] DIR3 | Creates the output directory DIR3 that contains the DNA sequences of introns within each class |
| Optional settings | -w[ritetofile] DIR4 | Creates the directory DIR4 that contains human friendly output from assigning candidate sequences intron RNA sequence |
| Example of optional usage | ./generate_rna_sequences -si ../storage_/5/Both\ 5\'\ and\ 3\'\ Boundaries/all -so ../storage_new/6 -o ../system_new/RNA\ and\ DNA\ Sequences -w ../readable/RNA  Result: Evaluates data about possible boundaries, assigns boundaries, and divides candidates into categories of certainty and ambiguity. Human readable output is printed to ../readable/RNA. | |
| **group_candidates** | | |
| Standard command | ./group_candidates -si ../storage_new/6/Have\ Intron\ Boundaries/ -o ../system_new/ORF_Class_Alignments_Group_DNA_Alignments | |
| Usage | ./group_candidates -si\|storablein DIR1 -o[d\|utput] DIR2 [options] | |
| Mandatory settings | -si\|storablein DIR1 | A path to a directory DIR1 that stores files that are used as the input sequences for assigning the intron groups |
|  | -o[d\|utput] DIR2 | Creates the output directory DIR2 that contains alignments of each classes conserved ORF sequence |

| Optional settings | -w[ritetofile] DIR3 | Creates the directory DIR3 that contains human readable output of introns grouped into clusters of >95% identity |
| --- | --- | --- |
|  | -a[lign] | Aligns the DNA sequence of each group of 95% identity |
|  | -c[utoff] NUM | A number NUM that is the pair-wise distance (PROTDIST) used for determining the groups; the default distance is 0.061, which corresponds to ~95% identity for these introns |
| Example of optional usage | ./group_candidates ../storage_/6/Have\ Intron\ Boundaries -o ../system_new/ORF_Class_Alignments_Group_DNA_Alignments -w ../readable/Groups -a –c 0.065  Result: Groups of introns are assigned based on a distance of 0.065 units apart. The downloaded DNA sequence of each group is aligned and output to../system_new/ORF_Class_Alignments_Group_DNA_Alignments. Human readable output is printed to ../readable/Groups. | |
| **select_prototypes** | | |
| Standard command | ./select_prototypes -si ../storage_new/6/Have\ Intron\ Boundaries/ -so ../storage_new/7 -o ../system_new/prototype_files | |
| Usage | ./select_prototype -si\|storablein DIR1 -so\|storableout DIR2 -o[utdir\|d] DIR3 | |
| Mandatory settings | -si\|storablein DIR1 | A path to a directory DIR1 that stores files that are used as the input sequences for assigning a prototype |
|  | -so\|storableout DIR2 | Creates the output directory DIR2 that stores files sorted into subdirectories from assigning a prototype |
|  | -o[d\|utdir] DIR3 | Creates the output directory DIR3 that contains the gathered information for all candidates and the prototype candidates |
| Optional settings | -w[ritetofile] DIR4 | A path to a directory containing human readable output |
|  | -l[ength] NUM | A number greater than 0 that is flanking sequence length included in the sequence 'dna_seq_with_flanks'; the default is 450 |
| Example of optional usage | ./select_prototypes -si ../storage_new/6/Have\ Intron\ Boundaries/ -so ../storage_new/7 -o ../system_new/prototype_files -w ../readable/Prototypes -l 500  Result: For each group of 95% identity, a prototype is selected, based on the longest pair-wise sequence match to its closest relative; if a known intron already belongs to the group, then it will be chosen as the prototype; the exon flanks included is 500 bp on each side of the predicted intron boundaries; human readable output is printed to ../readable/Prototypes. | |

**Additional file 2: Table S2. Output of each program**

| **blast_and_parse** | | | | | | | | | | | | |
| --- | --- | --- | --- | --- | --- | --- | --- | --- | --- | --- | --- | --- |
| Folder created: system_new | Subfolder created: blast_alignments | | | The folder contains one file per query, 22 files when using the default input. Each file contains the TBLASTN output for one search. | | | | | | | | |
|  | File created:  unique_candidate_hits.txt | | | Single file containing a list of GenBank accession numbers and coordinates for each hit. There were 3191 hits in July 2013. | | | | | | | | |
| **DNA_sequence_download** | | | | | | | | | | | | |
| In system_new: | File created:  sequences.txt | | | | | | | A single file containing all downloaded GenBank sequences (3191 sequence downloads in July 2013) | | | | |
|  | Subfolder created:  candidate_GenBank files | | | | | | | Files created:  bacterial_and_archaea.txt  chloroplast.txt  eukaryota.txt  mitochondrial.txt  unknown_organelle.txt  others.txt | | | | The same information as in “sequences.txt” but files are subdivided according to source. In July 2013, there were 2790 bacterial & archaebacterial, 370 eukaryotic, 343 organellar, 25 chloroplast, 308 mitochondrial, 27 unknown organellar, and 31 other sequences. Numbers do not add to 3191 because some sequences are included in more than one file. |
| **create_storage** | | | | | | | | | | | | |
| In system_new: | Subfolder created:  FASTA | | | | | | | Contains one fasta file per candidate intron, consisting of the DNA sequence of the RT match in the BLAST search | | | | |
| Folder created: storage_new | Subfolder created:  “0” | | | | | | | Contains one file for each candidate intron sequence (2790 files in July 2013). | | | | |
| **Filter_out_non_gpII_rts** | | | | | | | | | | | | |
| In system_new: | No change | | | | | | | | | | | |
| In storage_new: | Subfolder created:  “1” | | | Folders created:  All (2791), Probably Group II Intron (2754), Possibly Group II Intron (19), Probably Not Group II Intron (18) | | | | | | Each folder contains one file for each candidate intron in that category. Parentheses indicate the number of candidate sequences in each folder as of July 2013. | | |
| **find_intron_class** | | | | | | | | | | | | |
| In system_new: | No change | | | | | | | | | | | |
| In storage_new: | Subfolder created:  “2” | | | Folders created:  All (2753), Bacterial A (152), Bacterial B (195), Bacterial C (1007), Bacterial D (228), Bacterial E (140), Bacterial F (45), CL (561), ML (128), No hits (1), Unclassified (41), Undefined (256) | | | | | | Each folder contains one file for each candidate intron in that category. Parentheses indicate the number of candidate sequences in each folder as of July 2013. | | |
| **find_orf_domains** | | | | | | | | | | | | |
| In system_new: | No change | | | | | | | | | | | |
| In storage_new: | Subfolder created:  “3” | | | | Folders created:  All (2753), Normal ORF Domains (2160), Incomplete ORF Domains (504), Possible Tandem Intron-No ORF Domains Assigned (5), Possible Tandem Intron-ORF Domains Assigned (69), Possible Twintron (15) | | | | | | Each folder contains one file for each candidate intron in that category. Parentheses indicate the number of candidate sequences in each folder as of July 2013. | |
| **find_orf** | | | | | | | | | | | | |
| In system_new: | No change | | | | | | | | | | | |
| In storage_new: | Subfolder created:  “4” | | | | Folders created:  All (2160), Apparent Functional ORF (1822), FSPS in 0-X (222), Further Analysis (1), Multiple ORF Locations (25), ORF not Found (4), Possible ORF Problems (86). | | | | | | Each folder contains one file for each candidate intron in that category. Parentheses indicate the number of candidate sequences in each folder as of July 2013. | |
| **find_intron_boundaries** | | | | | | | | | | | | |
| In system_new: | No change | | | | | | | | | | | |
| In storage_new: | Subfolder created:  “5” | | Subfolders created: | | | | | | | | Each folder contains one file for each candidate intron in that category. Parentheses indicate the number of candidate sequences in each folder as of July 2013. | |
|  |  |  | All (1822) | | | | | | | |  |  |
|  |  |  | Both 5’ and 3’ Boundaries | | | All (1640) | | | | |  |  |
|  |  |  |  |  |  | High Probability (1003) | | | | |  |  |
|  |  |  |  |  |  | Medium Probability (520) | | | | |  |  |
|  |  |  |  |  |  | Low Probability (107) | | | | |  |  |
|  |  |  |  |  |  | Probably Tandem Intron (10) | | | | |  |  |
|  |  |  | No Boundaries (29) | | | | | | | |  |  |
|  |  |  | Only 5’ Boundary, | | | All (26) | | | | |  |  |
|  |  |  |  | | | High Probability (11) | | | | |  |  |
|  |  |  |  | | | Medium Probability (12) | | | | |  |  |
|  |  |  |  | | | Low Probability (3) | | | | |  |  |
|  |  |  | Only 3’ Boundary | | | All (127) | | | | |  |  |
|  |  |  |  | | | High Probability (61) | | | | |  |  |
|  |  |  |  | | | Medium Probability (52) | | | | |  |  |
|  |  |  |  | | | Low Probability (14) | | | | |  |  |
| **generate_rna_sequences** | | | | | | | | | | | | |
| In system_new: | Folder created:  Class_RNA_and_DNA_Sequences | | | | Folders created:  DNA Sequence  RNA Sequence | | Each folder contains one fasta file per class of DNA or RNA sequences. The sequences are not aligned. The DNA and RNA sequences differ only by T’s and U’s. | | | | | |
| In storage_new: | Subfolder created:  “6” | | | | Subfolders created:  All (1640), Have Intron Boundaries (1494), Ambiguous Intron Boundaries (146) | | Each folder contains one file for each candidate intron in that category. Parentheses indicate the number of candidate sequences in each folder as of July 2013. | | | | | |
| **group_candidates** | | | | | | | | | | | | |
| In system_new: | Folder created:  ORF_Class_Alignments_Group_DNA_Alignments | | | | Folders created:  Alignment of Each Class Conserved ORF | | | | | | Contains one “.aln” file per class with amino acid alignments for the IEP, but only for the “conserved” regions of domains 0-7 and X | |
|  |  |  |  |  | FASTA of Each Class Conserved ORF | | | | | | Contains one unaligned fasta “.fsa” file per class, and also a tree file “.dnd” made from the alignment | |
| In storage_new: | No change | | | | | | | | | | | |
| **select_prototypes** | | | | | | | | | | | | |
| In system_new: | Folder created:  Prototype_files | Folder created:  All Candidate Output Information | | | Collected&sorted features | | | | All Gathered Information | | | Contains one text file per class with all gathered information for each intron, concatenated |
|  |  |  |  |  |  |  |  |  | Genbank Entry | | | Contains one folder per class, with one text file per intron |
|  |  |  |  |  |  |  |  |  | Intron DNA Sequence | | | Contains one text file per class in fasta format |
|  |  |  |  |  |  |  |  |  | Intron IEP Sequence | | | Contains one text file per class in fasta format |
|  |  |  |  |  |  |  |  |  | Intron RNA Sequence | | | Contains one text file per class in fasta format |
|  |  |  |  |  | One folder per candidate intron | | | | Folders:  Bacterial A, Bacterial B, Bacterial C, Bacterial D, Bacterial E, Bacterial F, CL, ML, Unclassified, Undefined | | | Contains one folder for each candidate intron with five files containing information only for that intron: All Gathered Information, Genbank Entry, Intron DNA Sequence, Intron IEP Sequence, Intron RNA Sequence |
|  |  | Folder created:  Prototype Output Information | | | Collected&sorted features | | | | All Gathered Information | | | Contains one text file per class with all gathered information for each intron, concatenated |
|  |  |  |  |  |  |  |  |  | Genbank Entry | | | Contains one folder per class, with one text file per intron |
|  |  |  |  |  |  |  |  |  | Intron DNA Sequence | | | Contains one text file per class in fasta format |
|  |  |  |  |  |  |  |  |  | Intron IEP Sequence | | | Contains one text file per class in fasta format |
|  |  |  |  |  |  |  |  |  | Intron RNA Sequence | | | Contains one text file per class in fasta format |
|  |  |  |  |  | One folder per candidate intron | | | | Folders:  Bacterial A, Bacterial B, Bacterial C, Bacterial D, Bacterial E, Bacterial F, CL, ML, Unclassified, Undefined | | | Contains one folder for each candidate intron with five files containing information only for that intron: All Gathered Information, Genbank Entry, Intron DNA Sequence, Intron IEP Sequence, Intron RNA Sequence |
|  |  | File created:  Candidates from Selected Groups.txt | | |  | | | | | | | List of assigned groups and group members |
|  |  | File created:  Prototypes.txt | | |  | | | | | | | Single text file containing all information for each prototype intron |
| In storage_new: | Subfolder created:  “7” | | | | Subfolders created:  All (572), Bacterial A (10), Bacterial B (68), Bacterial C (180), Bacterial D (56), Bacterial E (27), Bacterial F (12), CL (172), ML (45), Unclassified (1), Undefined (1). | | | | | | Each folder contains one file for each candidate intron in that category. Parentheses indicate the number of candidate sequences in each folder as of July 2013. | |
